# Transcriptomic analysis of metabolic function in the giant kelp, *Macrocystis pyrifera*, across depth and season

**DOI:** 10.1111/nph.12160

**Published:** 2013-03-13

**Authors:** Talina Konotchick, Christopher L Dupont, Ruben E Valas, Jonathan H Badger, Andrew E Allen

**Affiliations:** J. Craig Venter Institute10355 Science Center Drive, San Diego, CA, USA

**Keywords:** comparative genomics, light harvesting complex, M*acrocystis pyrifera* (giant kelp), Phaeophyceae, quantitative PCR, RNA-Seq, transcriptomics, water-column gradients

## Abstract

- To increase knowledge of transcript diversity for the giant kelp, *Macrocystis pyrifera*, and assess gene expression across naturally occurring depth gradients in light, temperature and nutrients, we sequenced four cDNA libraries created from blades collected at the sea surface and at 18 m depth during the winter and summer.
- Comparative genomics cluster analyses revealed novel gene families (clusters) in existing brown alga expressed sequence tag data compared with other related algal groups, a pattern also seen with the addition of *M. pyrifera* sequences.
- Assembly of 228 Mbp of sequence generated *c*. 9000 isotigs and *c*. 12 000 open reading frames. Annotations were assigned using families of hidden Markov models for *c*. 11% of open reading frames; *M. pyrifera* had highest similarity to other members of the Phaeophyceae, namely *Ectocarpus siliculosus* and *Laminaria digitata*.
- Quantitative polymerase chain reaction of transcript targets verified depth-related differences in gene expression; stress response and light-harvesting transcripts, especially members of the LI818 (also known as LHCSR) family, showed high expression in the surface compared with 18 m depth, while some nitrogen acquisition transcripts (e.g. nitrite reductase) were upregulated at depth compared with the surface, supporting a conceptual biological model of depth-dependent physiology.

## Introduction

The brown algae (Class Phaeophyceae) are morphologically and geographically diverse. The multicellular Phaeophyceae range from microscopic filaments to the largest alga on earth, the giant kelp, *Macrocystis pyrifera*, reaching tens of meters in length. *Macrocystis pyrifera* is a dominant space competitor on temperate rocky reefs and provides a three-dimensional structure that supports many fish and invertebrate species (Dayton, 1985). Throughout its large geographic range, *M. pyrifera* is exposed to depth-dependent gradients of light, temperature, and nitrate conditions that vary depending on multiple temporal and spatial scales. Single individuals spanning the water column may experience seasonal to hourly variations in these conditions (Dean, 1985; Konotchick *et al*., 2012). Despite reaching great heights, *M. pyrifera* does not possess a vascular system for metabolite transport as plants do. Instead, organic material (i.e. mannitol and amino acids) is transported via specialized conducting cells with large pores called sieve tube cells (Parker, 1966; Manley, 1983). *Macrocystis pyrifera* may balance internal carbon and nitrogen through mannitol transport downward and amino acid transport upwards, especially under limiting conditions such as during typical southern California summer stratified water column conditions when upper parts of the kelp are not exposed to nitrate (Colombo-Pallotta *et al*., 2006).

The photosynthetically available light for kelp is greatest at the surface and exponentially decreases with depth with *c*. 1% of surface light levels typically reaching 20 m in the kelp forest (Gerard, 1984). Canopy shading by the kelp itself and particulate matter (both organic and inorganic) negatively affect light penetration, leading to decreased carbon fixation and growth by *M. pyrifera* blades (Towle & Pearse, 1973; Dean, 1985; Wing *et al*., 1993). While low light limits photosynthesis, excess light can damage photosystem II, leading to oxidative stress. In response, algae have adaptations to optimize light absorption under variable light availability through differential expression of light-harvesting complexes, some of which are involved in both light capture and photoprotection (Savard *et al*., 1996; Peers *et al*., 2009).

Biological processes in kelp affected by nitrate include recruitment, growth, survivorship, reproductive output and stress tolerance (Mann, 1973; Jackson, 1977; Zimmerman & Kremer, 1986). Thus, the amount of nitrate exposure can have a large effect on the condition and the existence of a kelp bed. There is a strong linear relationship between nitrate concentration and temperature (°C) in the southern California nearshore at temperatures below 14.5°C, with colder waters possessing more nitrate (Kamykowski & Zentara, 1986; Dayton *et al*., 1999; Konotchick *et al*., 2012); at temperatures above 14.5°C, nitrate concentrations are not generally detectable. Seasonally, in southern California, surface nitrate concentrations are low for most of the year but increase during the winter when the water column is well mixed or upwelling occurs (Jackson, 1977).

Kelps respond physiologically on different temporal and spatial scales to their physical environment. For example, during summer months and El Niño years, temperatures rise, nutrients decrease and ultimately kelp densities decrease (Jackson, 1997; Dayton *et al*., 1999). Outward phenotypic signs of stress may include sloughing of tissue, increased epiphytic growth, disease and ultimately senescence. These are the final result of the sum of kelp physiological responses to stressors over an integrated amount of time (e.g. months to years). Physiological responses of *M. pyrifera* on much shorter time-scales (i.e. time scales of minutes to hours), such as gene expression responses to vertical nitracline shifts of 10 m or more in a few hours (Konotchick *et al*., 2012), before these conspicuous phenotypic changes manifest is unknown.

Genomic information is sparse for macroalgae (Jamers *et al*., 2009) and for the Phaeophyceae in particular (Phillips *et al*., 2008). *Macrocystis* and the Phaeophyceae belong to the Stramenopile lineage which is thought to have diverged from other major eukaryotic groups over a billion years ago (Douzery *et al*., 2004; Yoon *et al*., 2004). As a result, unique metabolic and developmental features have evolved (Cock *et al*., 2010a,b; Michel *et al*., 2010). Phaeophytes are evolutionarily distant from other photoautotrophs, thus the applicability of knowledge learned from terrestrial model species such as the plant *Arabidopsis* remain somewhat limited for this group (Peters *et al*., 2004; Keeling *et al*., 2005). Recognizing the need for model brown algal species, researchers sequenced the genome of the filamentous brown alga *Ectocarpus siliculosus* (Peters *et al*., 2004; Waaland *et al*., 2004; Charrier *et al*., 2008; Cock *et al*., 2010b). In brown algae, expressed sequence tags (ESTs) have been used to identify specific genes and expression patterns involved in various processes such as specific life stages or various experimentally manipulated conditions such as stressful light or temperature (Crepineau *et al*., 2000; Roeder *et al*., 2005), determine phylogenetic analysis/evolutionary relationships (Phillips *et al*., 2008; Nam *et al*., 2011), investigate carbon metabolism and develop sequence-based molecular toolkits (Moulin *et al*., 1999).

The current increasing trend in genomic information in the marine realm provides an opportunity to expand our knowledge of the Phaeophyceae. Decreased sequencing costs and an increase in sequencing capabilities make it possible to examine species beyond the typical or traditional model and laboratory organisms. A major assumption of comparative biology is that the more closely two organisms are related, the more they will share molecular, biochemical and morphological features (Keeling *et al*., 2005). Thus, the presence of both single-celled and multicellular heterokont genomes (i.e. the diatoms *Phaeodactylum tricornutum*, *Thalassiosira pseudonana* and *Aureococcus anophagefferens* and the phaeophyte *Ectocarpus siliculosus*) provides phylogenetic context, potential model systems for detailed functional information and a new opportunity for comparative genomics and gene discovery in this group (Armbrust *et al*., 2004; Bowler *et al*., 2008; Cock *et al*., 2010b; Gobler *et al*., 2011).

In order to investigate gene expression in the giant kelp and make tangible functional links to its closest relatives we needed species-specific data. For example, while the canonical pathways for nitrate uptake, reduction and incorporation into proteins are well known, the gene models and transcript sequences for these important enzymes in *M. pyrifera* are not. In this study, we use a pyrosequencing-based RNA-Seq approach to investigate the transcripts from four *M. pyrifera* libraries spanning the water column and seasons. Objectives of this study were to: increase the number of annotated transcriptional units (TUs) for *M. pyrifera*, develop sequence-based tools for ecophysiological study, and examine physiological patterns in response to light, temperature and nitrate, with depth.

## Materials and Methods

### *Macrocystis pyrifera* sample collection

Blade tissue was sampled from *M. pyrifera* (Linnaeus) C. Agardh individuals in La Jolla, California, USA (32°51.0 N; 117°17.5 W) on 7 January 2009 and 31 July 2009 using SCUBA. On each date, pieces of blade tissue were collected at the sea surface (0 m) and at 18 m depth, along the same stipe, for a total of four pieces of blade tissue used for transcriptomic library preparation. Surface blades were collected at least 1 m away from the apical growing region and the sampling at 18 m avoided the reproductive sporophylls near the base of an individual so that only the sporophyte generation was sampled. For consistency and to minimize within-blade variability, all samples were collected near the base of each blade. Any wounding effects were assumed to be consistent across samples. At the end of each dive, blades were immediately cleaned of any visible epiphytes by scrubbing with 100% ethanol and cheesecloth and then frozen on dry ice for transport to the laboratory.

To measure temperature changes and to infer nutrient concentration, thermistor chain data were collected at 10-min intervals using TidBit temperature data loggers with *c*. 0.2° resolution, and *c*. 5 min response time (Onset, Bourne, MA, USA). TidBits were placed on the bottom (located in 22 m water depth) and at 2, 6, 10, 14 and 18 m above the bottom (mab).

### Library construction

RNA extraction followed a modified protocol (Apt *et al*., 1995). Frozen algal tissue was ground to a powder on liquid nitrogen then added to an extraction buffer (100 mM Tris–HCl pH 8.0, 1.5 M NaCl, 20 mM ethylenediaminetetraacetic acid (EDTA), 20 mM DTT and 2% cetyltrimethylammonium bromide (CTAB)) at 1 : 1 w : v ratio and mixed at room temperature (RT) for 15 min then heated to 65°C for 20 min. This was followed by a ½-volume chloroform extraction, 5 min at RT, centrifugation at 10 000 ***g*** for 30 min at 4°C and collection of the supernatant. Addition of 1/3 volume ethanol was used to precipitate polysaccharides, which was followed by a second chloroform extraction. 3.0 M LiCl and 10% v : v β-mercaptoethanol was added to the aqueous phase and placed at −20°C overnight. RNA was precipitated by centrifugation at 14 000 ***g*** for 30 min at 4°C and followed by two 75% ethanol washes before resuspension.

Total RNA was cleaned using RNeasy mini Kit and the optional DNase digestion (Qiagen). RNA was amplified using MessageAmp II Kit (Ambion) with a second round of amplification. Single-strand cDNA was synthesized using superscript III (Invitrogen, Carlsbad, CA, USA) and oligo(dT) primers, then cleaned using RNAClean to remove salts, unincorporated primers and dNTPs (Agencourt, Beckman Coulter Genomics, Beverly, MA, USA). CloneMiner kit (Invitrogen) was used to synthesize second-strand cDNA and cleaned with AMPure (Agencourt). Size-selected cDNA (0.5–1 kb) from the four libraries was purified using QiaQuick gel extraction kit (Qiagen). A high-sensitivity DNA Assay chip was used to assess quality (Agilent, Santa Clara, CA, USA).

### *De novo* transcriptome assembly and annotation analysis

Each of the four libraries was sequenced using pyrosequencing technology (454 Life Sciences, Roche, Branford, CT, USA). Reads were filtered using cd-hit-454 (Teal & Schmidt, 2010) and assembled using newbler 2.3 (454 Life Sciences). Open reading frames (ORFs) were called using fraggenescan (Rho *et al*., 2010) and functionally annotated for pfams and tigrfams using hidden Markov model (HMM) searches (*e*-value cut-off 1e^−5^). Kegg orthologs (KO) annotations resulted from blast against Kegg (*e*-value cutoff 1e^−5^; v. 20110830). Phylogenetic annotation was accomplished using an internal JCVI database, PhyloDB, which contains all completed and draft algal genomes as well as all Phaeophyceae EST libraries available in NCBI GenBank for annotation. Differential expression of ORFs was evaluated using an empirical Bayes method (Wu *et al*., 2010).

### Quantitative (q)PCR analysis of gene transcription

For each sample, 1 μg RNA was purified using RNeasy (Qiagen) and 200 ng was reverse transcribed using QuantiTect (Qiagen) and diluted 50 fold. RNA for qPCR came from the same extraction used to create the transcriptomic libraries. The PCR products were quantified from the same batch of cDNA to minimize experimental variation caused by the cDNA synthesis process. Samples were run on a 7900HT Fast Real-Time PCR system and 7500 Fast Real-Time PCR systems (Applied Biosystems, Carlsbad, CA, USA). Using the annotations of the *M. pyrifera* ESTs to identify target TUs, we created primers for a variety of potential housekeeper genes and genes of interest (see the Supporting Information Table S1; Le Bail *et al*., 2008). After exclusion of those with poorly amplified products, 16 potential reference genes were evaluated to select the most stable reference genes (Vandesompele *et al*., 2002). We used eukaryotic initiation factor 2 alpha subunit (IF2A) and a protein required for 18S rRNA maturation and 40S ribosome biogenesis (18Smat) for housekeeper genes based on this analysis and their *C*_t_ range. The majority of candidate *M. pyrifera* sequences were aligned with the corresponding *E. siliculosus* sequence and primers were created in the region of overlap using primer 3 (Rozen & Skaletsky, 2000) with a target length of 100–150 bp and a max *T*_m_ difference < 8°C. We quantified expression levels using quantitative real-time PCR via the delta-delta cycle threshold method (ΔΔ*C*_t_) and reported the data as fold-change comparisons between the surface and 18 m depth; significance was assessed using a two-tailed Welch's *t*-test, df = 2, *P* ≤ 0.05.

### Comparative genomics of available Phaeophyceae data with other algal groups

To comparatively assess the currently available Phaeophyceae genomic repertoire and the addition of *Macrocystis pyrifera* dataset, we clustered sequences using the OrthoMCL algorithm, as it was reviewed as exhibiting the best overall balance between sensitivity and specificity for eukaryotic orthology detection, which can be difficult because of the complex protein domain structure in eukaryotes (Li *et al*., 2003; Chen *et al*., 2007). The species included in each group in these analyses are listed in Table S2.

## Results

### Transcriptomic coverage and annotation results

**Fig. 1:**
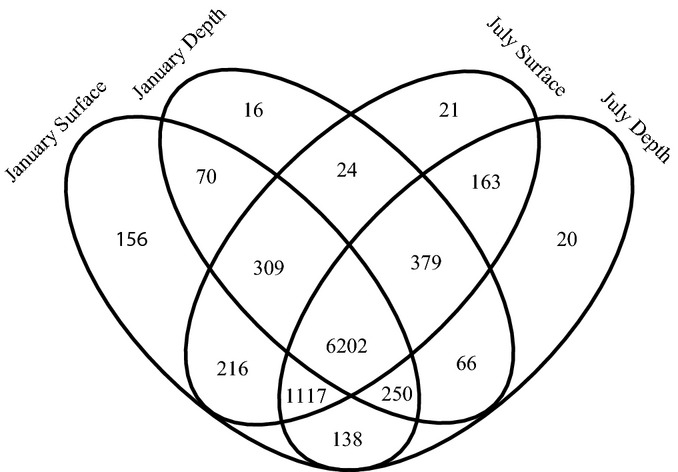
Isotig Venn diagram showing the distribution of isotigs between the four *Macrocystis pyrifera* libraries (January surface, January depth, July surface, July depth).

Sequences from four transcriptomic libraries totaling 228 Mbp yielded an average of 118 000 reads per library after filtering artificial replicates (Gomez-Alvarez *et al*., 2009), an average read length of 323 bp and a GC content of 48% (Table 1). The GC content falls within the range of reported values of 39.9–54% for the Phaeophyceae (Le Gall *et al*., 1993). Reads (71% of total) were assembled *de novo* into 9147 isotigs, or transcript variants, with an N50 size of 817 bp. The average isotig size was 642 bp with a maximum of 2872 bp. For comparison, *E. siliculosus*, a small filamentous intertidal brown alga, has *c*. 16 000 genes, of which 9601 had EST support from six cDNA libraries corresponding to different developmental stages and growth conditions (Cock *et al*., 2010b). We found 67.8% of isotigs in all four libraries (Fig. 1), indicating a large active transcriptional core across environmental gradients, providing confidence in the *de novo* assembly and in the ability to examine expression differences in transcripts common to all libraries. Only 2.3% of isotigs were only found in one library (Fig. 1).

**Table 1:** Pyrosequencing statistics and annotation results

Transcriptome characteristics	Value
Sequence data (Mbp)	228
Total number of reads (× 1000)	704
Schmidt filtered reads (× 1000)	472
Number of libraries	4
Mean length of filtered reads (bp)	323
Percentage of GC	48
Total isotigs	9147
N50 isotig size (bp)	817
Number of open reading frames	11 844
Percentage of open reading frames with a pfam annotation	5.6
Percentage of open reading frames w/PhyloDB taxon ID	11.2
Percentage with best match to *Ectocarpus siliculosus*^1^,^2^	72.6
Percentage with best match to *Laminaria digitata*^1^,^3^	11.3
Percentage with best match to other Phaeophyceae^1^,^4^	6.8

1 Percentage of PhyloDB annotated ORFs.

2 67 106 *E. siliculosus* entries in database.

3 3124 *L. digitata* entries in database.

4 15 230 other Phaeophyceae entries in database. These include expressed sequence tags (ESTs) from *Fucus serratus*, *Fucus vesiculosus*, *Sargassum binderi*, and *Saccharina japonica*.

Of 11 844 ORFs called, 5.6% had HMM-derived pfam annotations (Table 1); 11.2% of ORFs had taxon ID matches in PhyloDB and *E. siliculosus*, the only phaeophyte with a sequenced genome, had the highest percentage of matches (72.6%). The next highest percentage of best matches was to *Laminaria digitata* (11.3%). There is about *c*. 20-fold less genomic data available for *L. digitata* than there is for *E. siliculosus* (Table 1); however, *L. digitata* is taxonomically related to *M. pyrifera* at the family level, while *E. siliculosus* is related at a class level. The similarity of orthologous proteins between *M. pyrifera* and *L. digitata* and *E. siliculosus* was 86.8% and 79.5%, respectively (Fig. S1).

### Comparative analysis of available Phaeophyceae sequence-based data

**Fig. 2:**
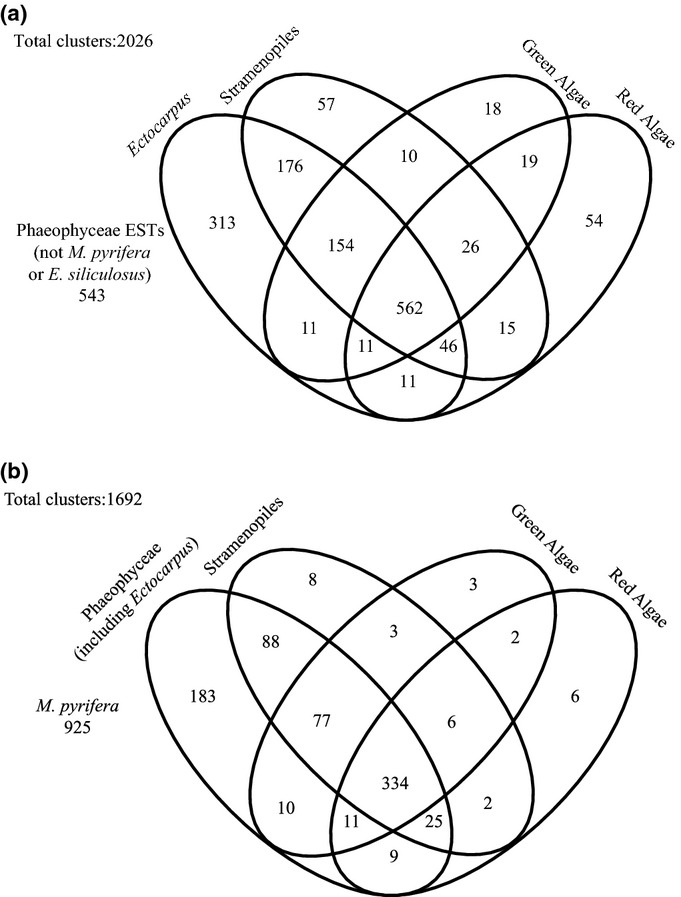
Distribution of clusters of open reading frames (ORFs) for (a) Phaeophyceae expressed sequence tags (ESTs; not including *Macrocystis pyrifera*), with ORF clusters from *Ectocarpus siliculosus*, stramenopiles, green algae and red algae, and (b) all EST-derived *Macrocystis pyrifera* ORF clusters with the ORF clusters from Phaeophyceae ESTs (including *Ectocarpus siliculosus*), the stramenopiles, green algae and red algae. The genomes and EST libraries used in this analysis are listed in the Supporting Information, Table S2. Annotations (when available) for clusters on the outside of the Venn are listed in Table S3.

Open reading frame-level clustering as a preliminary analysis of phaeophyte protein diversity reveals novel clusters distinct from their closest relatives (Fig. 2). To assess the level of Phaeophyceae sequence data to date, we first compared EST sequences from the Phaeophyceae (not including *M. pyrifera* sequences) with the *Ectocarpus* genome, Stramenopiles, green algae and red algae (Fig. 2a, Table S2). Phaeophyceae ESTs grouped into 2026 clusters, 543 of which were unique to Phaeophyceae ESTs (exclusive of *Ectocarpus* and *Macrocystis*) and not shared with *Ectocarpus*, Stramenopiles, green algae or red algae; annotations for these clusters are listed in Table S3. Five hundred and sixty-two clusters were shared between all groups; examples of these shared annotations include ubiquitin, ribosomal proteins, ATP synthases and photosynthesis-related genes. Phaeophyceae ESTs also shared a large number of clusters with only *Ectocarpus* (313), and with clusters shared by *Ectocarpus* and Stramenopiles (176).

We then compared the addition of the *M. pyrifera* EST data generated in this study. In this analysis, the Phaeophyceae includes all the brown algal ESTs and the *Ectocarpus* genome (Fig. 2b). *Macrocystis pyrifera* ORFs were grouped into 1692 clusters, 925 of which were only found in *M. pyrifera* and not in the other groups; none of these clusters had an annotation (Table S3). The distributions with the highest numbers of clusters were shared with all groups (334), shared with only the brown algae (183) and shared with only the brown algae and the Stramenopiles (88). Comparison of the *M. pyrifera* clusters with only the brown algae ESTs (and not the *Ectocarpus* genome) in a similar Venn analysis is shown in Fig. S2 and Table S3. Novel clusters from Figs 2 and S2 with differential expression patterns are also indicated in Table S3. Comparisons of the phaeophyte sequences generated to date (including this study) indicate a high level of novel protein discovery in this group.

### Physiological patterns in transcript profiles

**Fig. 3:**
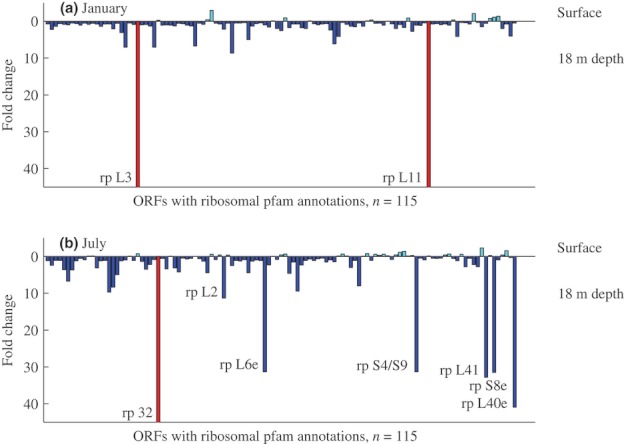
Log_2_ fold change comparison based on the library normalized transcriptomic read data between the surface and depth for open reading frames (ORFs) with ribosomal pfam annotations and with total read counts of at least 10 across all libraries and at least one read in each library (*n* = 115) for (a) January and (b) July. Bars are ordered by summed counts across all libraries (from 713 to 10). Teal bars had higher expression at the surface and blue bars had higher expression at depth. Red indicates values that exceed the axis. Annotations are shown for ribosomal proteins with a fold-change > 10; rp, ribosomal protein.

Many of the top ORF pfam descriptions (in terms of normalized summed read counts across all libraries) were to ribosomal proteins. A fold-change comparison of ORFs with ribosomal pfam descriptions between surface and depth for both seasons is shown in Fig. 3, indicating a high level of protein synthesis at depth. Top nonribosomal ORF pfams included a photosynthetic reaction center protein, chaperonin, ATP synthase and heat shock protein; for a more complete list see Table 2 (top 30) and Table S4 (top 31–100). Taxon matches were to mainly to Phaeophyceae members and to a few diatom species. Patterns between surface and depth emerged through differential comparison of expression with each season (Table S5). Transcripts upregulated in the surface included those encoding photosynthesis and damage repair proteins (e.g. photosynthetic reaction center protein, chlorophyll-binding protein, Hsp 70 protein, PsbP), while at depth ribosomal proteins dominated.

**Table 2:** The top 30 open reading frames (ORFs) with non-ribosomal pfam annotation ranked by total reads across the four libraries, the pfam description, the best species match and significant differential expression patterns in January and July

Sum	pfam description	Taxon	January^1^	July^1^
353	Photosynthetic reaction centre protein	*Odontella sinensis*	+++	+
327	Chaperonin 10 kDa subunit	*Laminaria digitata*		
325	ATP synthase	*Ectocarpus siliculosus*	++	
312	Hsp70 protein	*Synechococcus* sp. PCC 7002		
304	3-beta hydroxysteroid dehydrogenase/isomerase family	*Ectocarpus siliculosus*		
224	Elongation factor 1 gamma, conserved domain	*Ectocarpus siliculosus*		
210	Excisionase-like protein||Bacteriophage lambda integrase, *N*-terminal domain	*Phaeocystis globosa*	+++	
210	LSM domain	*Ectocarpus siliculosus*		
182	Chlorophyll *A*–*B* binding protein	*Fucus serratus*	+++	+
147	NifU-like domain	*Ectocarpus siliculosus*		
146	Protein of unknown function (DUF1077)	*Ectocarpus siliculosus*		
143	Ubiquitin family	*Ectocarpus siliculosus*		
137	GcpE protein	*Fucus vesiculosus*	+	
136	ATP synthase subunit D	*Ectocarpus siliculosus*	+	
134	RNA polymerase Rpb3/Rpb11 dimerization domain	*Fucus serratus*	+	
132	Elongation factor Tu GTP-binding domain	*Ectocarpus siliculosus*	−	++
132	Phosphotyrosyl phosphate activator (PTPA) protein	*Ectocarpus siliculosus*		
109	DNA-directed RNA polymerase, 7 kDa subunit	*Fucus vesiculosus*	−	
109	WD domain, G-beta repeat	*Ectocarpus siliculosus*		
108	TATA-binding related factor (TRF) of subunit 20 of mediator complex	*Ectocarpus siliculosus*		
107	Cytosol aminopeptidase family, catalytic domain	*Ectocarpus siliculosus*		
99	Vacuolar (H+)-ATPase G subunit	*Ectocarpus siliculosus*		
98	Double-stranded DNA-binding domain	*Ectocarpus siliculosus*		
97	Nucleoside diphosphate kinase	*Laminaria digitata*		+
95	Domain of unknown function (DUF1982)	*Ectocarpus siliculosus*		
93	Pyridine nucleotide-disulphide oxidoreductase	*Ectocarpus siliculosus*		
84	Proteasome subunit	*Ectocarpus siliculosus*		
83	Eukaryotic porin	*Ectocarpus siliculosus*		+
81	TspO/MBR family	*Ectocarpus siliculosus*		
78	AMP-binding enzyme	*Ectocarpus siliculosus*		

1 Plus symbols indicate higher expression in the surface; minus symbols indicates higher expression at depth. The highest log-fold supported by a probability of 0.95 or greater is shown by the number of symbols; one symbol equals a fold-change between 0.5 and 1.5, two symbols equals a fold-change between 1.5 and 3.0, and three symbols equals a fold change > 3.0.

**Fig. 4:**
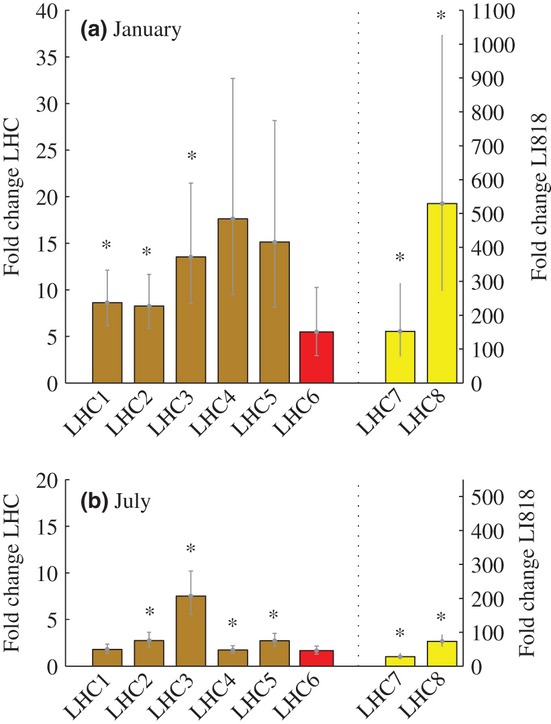
Quantitative PCR fold-change expression differences between the surface and at 18 m depth for light-harvesting complexes (LHC) found in *Macrocystis pyrifera*. LHCs assigned to FCP are shown in brown, to red shown in red and to LI818 shown in yellow (Table S6). The fold-change of the LI818 LHCs (yellow bars) was greater than the other LHCs (brown or red) in both (a) January and (b) July, (note different *y*-axis scales). Error bars represent standard deviation and asterisks indicate significant differences (two-tailed Welch's *t*-test, df = 2, *P* ≤ 0.05).

Light-harvesting genes were examined because their ORF counts showed differences between libraries in the pyrosequencing transcriptome and there is more knowledge about these genes than other genes in the Phaeophyceae class (Green *et al*., 1991; Green & Durnford, 1996; Dittami *et al*., 2010). blast searches of the transcriptome assembly identified eight contigs coding for putative light-harvesting complex (LHC) proteins (Table S6). For comparison, 53 LHCs were found in the genome of *E. siliculosus* (Dittami *et al*., 2010). A phylogenetic analysis of the *M. pyrifera* LHC and the LHC from other heterokonts grouped the *M. pyrifera* LHC isotigs into canonical LHC groups FCP, LHCR and LHCSR (Table S6). In both qPCR experiments across depth gradients, most *Mp*LHC showed significantly greater qPCR expression at the surface than at depth (up to 530 fold change; Fig. 4).

**Fig. 5:**
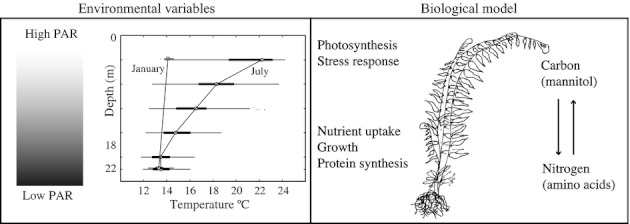
A schematic illustrating the gradients in light and temperature (and by proxy nutrients) that an individual *Macrocystis pyrifera* may span. Temperature inset is a boxplot of 10-min sampling interval data for the 2 wk leading up to the day of collection in January 2009 and July 2009 in La Jolla (CA, USA). On the right, is a conceptual model of dominant physiological processes with depth seen in the transcript counts and quantitative PCR validation and the major transport materials found in sieve tube sap.

**Fig. 6:**
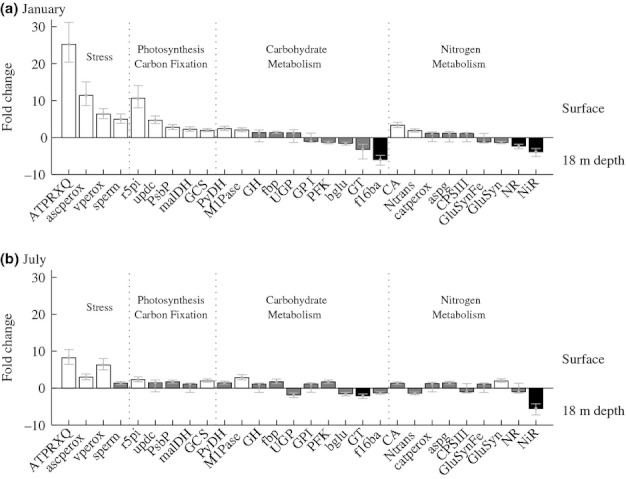
Quantitative PCR fold-change expression differences between the surface (above 0) and 18 m depth (below 0) for (a) January 2009 and (b) July 2009. Open bars, significant expression in the surface vs depth; closed bars, significant expression at depth vs the surface; tinted bars, non-significant values (two-tailed Welch's *t*-test, df = 2, *P* ≤ 0.05). Error bars represent standard deviation. The functional descriptions and primer sets for each transcriptional unit are listed in Table S1.

Because of the large environmental differences in light and temperature/nutrient conditions with depth (Fig. 5), we evaluated various stress response, photosynthesis, carbon metabolism and nutrient uptake genes using qPCR (Table S1). Antioxidant genes showed patterns of higher expression at the surface during both seasons for a protein of the periredoxin group (ATPRX_Q), l-ascorbate peroxidase and vanadium bromoperoxidase (Fig. 6). ATPRX_Q showed the greatest fold-change in the surface compared with depth in both seasons. Two photosynthesis/carbon fixation genes showed higher surface expression in both seasons: ribose-5-phosphate isomerase (r5pi), which is involved in the pentose phosphate pathway and produces sugars and NADPH, and the glycine decarboxylase complex (GCS). In January, several other photosynthesis-related genes showed upregulation in the surface: uroporphyrinogen decarboxylase (involved in porphyrin and chlorophyll metabolism), PsbP, the photosystem II oxygen evolution complex protein required for PSII to be fully operational, and malate dehydrogenase. Mannitol-1-phosphate 5-dehydrogenase, involved in metabolism of mannitol, one of the main storage carbohydrates in *M*. *pyrifera*, was upregulated in the surface in both seasons. Spermidine/spermine synthase, a regulator in stress-signaling pathways (Kasukabe *et al*., 2004), showed higher expression in the surface in January. By contrast, nitrite reductase, the enzyme catalysing the conversion of nitrite into ammonia, showed higher expression at depth in both seasons. Nitrite reductase is usually regulated in coordination with nitrate reductase (catalyses the nitrate → nitrite conversion), as was the case in the January libraries.

## Discussion

### Contributing to the genomic knowledge of the Phaeophyceae

The presence of ‘novel’ gene families in Phaeophyceae is supported by existing and new data (Figs 2, S2, Table S3). The sequences generated in this study clustered more often with the most closely related groups (Figs 2b, S2). The low level of pfam annotation (5.6%) and taxon ID match (11.3%) in our *de novo* assembly might suggest spurious gene discovery. However, the detection of the majority of isotigs in all libraries (Fig. 1), high identity level to other Phaeophyceae members (Fig. S1) and GC content in the range expected for Phaeophyceae suggests otherwise. Low annotation percentages based on blast approaches for brown algal ESTs have been seen before (i.e. 6.5% for *Choristocarpus*; Phillips *et al*., 2008). In addition, transcripts with assigned annotations and examined with qPCR showed patterns consistent with their environmental context (discussed below). Knowledge remains limited because the majority of genes (including those in novel clusters) cannot be assigned a function through traditional blast-based approaches. Linking ecological and physiological studies with gene expression data in an iterative manner can improve future annotations. Specifically, the categorization of unannotated clusters could serve as a basis for comparison with other populations on the California coast that experience different physical regimes. Insights from comparative genomics will continue to improve with the addition of more reference genomes and more annotated genes.

The transcripts from this study were collected from the blades of the *M. pyrifera* sporophyte generation. With additional cDNA sequencing of the gametophyte generation (Roeder *et al*., 2005), reproductive sporophylls, the apical growing region or other morphological parts, it is reasonable to expect that the number of annotated TUs can further expand the knowledge of the gene repertoire of *M. pyrifera*. Based on the high protein discovery seen thus far in the Phaeophyceae, is reasonable to assume that exploration of additional taxonomic members could yield insights into potentially unique evolutionary and physiological traits.

### Application of transcriptomic information to develop tools for ecological study

Our broad untargeted transcriptomic view allows us to begin to identify key mechanisms in the physiological and metabolic reactions to a variable environment. The identification of transcriptional units and development of primer sets for examining expression of select metabolic genes enables targeted hypothesis testing of physiological response to environmental conditions. Both transcriptional count data (Table S5) and quantitative PCR validation (Figs 4, 6) provide support for the hypothesis of Colombo-Pallotta *et al*. (2006) where there is localization of different metabolic processes occurring in different parts of the kelp (Fig. 5).

At the surface, where irradiance levels are highest and the potential for oxidative damage is most intense, physiological processes are focused on protection from the damaging effects of the sun as well as capture of light energy for photosynthesis. Several stress-related proteins showed higher qPCR expression levels at the surface in both seasons, including antioxidant proteins (i.e. ATPRX_Q) and peroxide proteins (i.e. l-ascorbate peroxidase and vanadium bromoperoxidase). Ascorbate peroxidases are associated with photo-oxidative stress in algae (Ishikawa & Shigeoka, 2008). Vanadium bromoperoxidases are found in several species of brown algae and are also thought to be involved in biotic and abiotic stress responses (La Barre *et al*., 2010). The intense and variable light environment at the surface upregulates stress-response genes.

Light appears to regulate gene expression strongly in *M. pyrifera*. The high expression at the surface of the LHCSR *Mp*LHCs is consistent with a role in photoprotection (e.g. nonphotochemical quenching, or NPQ) in a highly variable environment (Peers *et al*., 2009). The NPQ mechanisms in *M. pyrifera* differ from those of higher plants and may be linked with this LHC group (García Mendoza & Colombo-Pallotta, 2007; García Mendoza *et al*., 2011). Having multiple LHCs is thought to facilitate the ability to fine-tune control of photosynthesis electron flow for blades exposed to intermittent high and low irradiances at the surface. More than half of the light-harvesting complex genes showed significantly higher expression in the surface than at depth, with those in the LHCSR clade having the largest fold-change differences (Fig. 4). Other studies show functional differentiation between surface and basal blades in terms of photobiology, including enhanced photoprotection, higher maximum photosynthetic rate, higher photosystem II (PSII) electron transport rate, decreased pigment concentration and lower photosynthetic efficiency in surface blades compared with basal blades (Gerard, 1986; Smith & Melis, 1987; Colombo-Pallotta *et al*., 2006).

Deeper blades tend to be shaded by the canopy and are generally acclimatized to low light conditions. Physiological adaptations of *M. pyrifera* to gradients in light include higher relative amounts of chlorophyll and fucoxanthin and larger antennae sizes in blades found at 20 m compared with surface blades (Smith & Melis, 1987; Colombo-Pallotta *et al*., 2006). Blade relocation and canopy removal experiments have shown that differences in light use efficiency likely resulted from acclimatization to light conditions rather than age of blades (Gerard, 1986). The majority of photosynthesis occurs in the surface canopy with half of the standing crop lying between the surface and 1.5 m as fronds reaching and spreading along the surface (McFarland & Prescott, 1959; Towle & Pearse, 1973; Gerard, 1986). The daily increase in the dry weight of the growing tip (apical region) is greater than the products of its photosynthesis; in addition, blades further down the stipe have rates of photosynthesis that exceed their rate of growth, implying likely transport of materials (Sargent & Lantrip, 1952).

At the base of the kelp, where nutrient levels tend to be much higher than at the surface, especially during the stratified summer months, incorporation of nitrogen into amino acids and ultimately proteins may be a dominant process (Table S5). There was upregulation of nitrite reductase (NiR) at depth during both seasons, which catalyses the conversion of nitrite into ammonium (Fig. 6). *M. pyrifera* has the capacity for long-term storage of carbon and nitrogen in excess of immediate demands, which would allow for the decoupling of growth from immediate ambient conditions (Gerard, 1982). Thus, nutrient uptake may not only be dependent on current nutrient availability but also previous nutrient exposure. Although dissolved nitrate levels are typically elevated at depth, temperatures below 14.5°C occurred at both the surface and depth in January, suggesting the presence of biologically available levels of nitrate at both depths (Fig. 5). Expression of NR in January was higher at depth, perhaps indicating regulation by light (Dohler *et al*., 1995), elevated nutrient levels or other factors. Factors such as nutrient uptake metabolites, time of day, light and CO_2_ concentration can affect nutrient uptake, transcription and activity of nitrate reductase (Crawford *et al*., 2000). Increased dissolved inorganic nitrogen pools at depth could drive higher levels of protein synthesis, as suggested by upregulation of genes encoding ribosomal proteins and associated increases in cellular nitrogen demand and transcript levels for genes encoding proteins involved in nitrate assimilation.

It is difficult to say whether the individuals sampled in this study are representative of general patterns or what is driving the observed expression patterns without follow-up experiments of sufficient spatial and temporal coverage; these experiments are now possible using the reference transcriptome generated here. In addition, owing to our method of sampling along a stipe, surface blades are of younger age than the deeper blades, which may have an affect on gene expression; to decouple the effects of age with physiological response, blade translocation experiments could be conducted using these molecular tools. Finally, it is important to note that this study focused on transcriptomic data, and important insights from genomic or proteomic modifications are not addressed in this study.

Natural populations of *M. pyrifera* inhabit a variable physical environment and yet we were able to see strong gene expression patterns emerge. Differences in depth of dominant metabolic processes were apparent in these natural samples. Transcript levels were consistent with environment-regulated ecophysiology and this study provides a starting point for future studies coupling gene expression with finer-scale environmental measurements and other populations. Efforts to tease apart variables that co-vary together in nature (e.g. temperature and nitrate) can begin to be addressed using these tools. Through transcriptional profiling of the giant kelp, we have created a sequence-based tool kit for further physiological study of this species and demonstrate differences in metabolic function across environmental gradients with depth.

## Supporting Information

The sequences in this study have been deposited in NCBI's Sequence Read Archive (SRA) under the following accession numbers: SRX220272, SRX220273, SRX220275, and SRX220277, found in SRA Study SRP018250).

**Fig. S1** Percent similarity at the protein level between *Macrocystis pyrifera* and *Ectocarpus siliculosus* (blue) and *Laminaria digitata* (green) using BLAST for alignments with > 75% of the total read length.

**Fig. S2** Distribution of clusters of all EST-derived *Macrocystis pyrifera* ORFs with the brown alage split up by genus: *Laminaria, Fucus* (2 species), *Sargassum* and *Ectocarpus*. Annotations (when available) for clusters on the outside of the Venn are listed in Table S3.

**Table S1** *Macrocystis pyrifera* quantitative PCR targets, function, primer sets, and closest *Ectocarpus siliculosus* sequence match

**Table S2** Genomes and ESTs used in comparative cluster analysis and their sources

**Table S3** Non-redundant annotations for novel clusters in Figs 2 and S2

**Table S4** Continuation of Table 2: the top 31–100 open reading frames (ORFs) with non-ribosomal pfam annotation ranked by total reads across the four libraries, the pfam description, the best species match, and significantly differential expression patterns in January and July

**Table S5** Differentially expressed open reading frames (ORFs) with pfam annotation and summed read counts across the four libraries, the best species match, and significant differential expression patterns in January and July

**Table S6** Light-harvesting complexes identified from isotigs in *Macrocystis pyrifera*
