# Supplementary material for: Transcriptomic analysis of metabolic function in the giant kelp, Macrocystis pyrifera, across depth and season
Source: New Phytol. 2013 Mar 13;198(2):398–407. doi: 10.1111/nph.12160 (PMC3644879; doi:10.1111/nph.12160)
Supplement: Supplementary file 1 [file nph0198-0398-SD1.pdf]

Supporting Information

Fig. S1: Percent similarity at the protein level between *Macrocystis pyrifera* and *Ectocarpus siliculosus* (blue) and *Laminaria digitata* (green) using BLAST for alignments with >75% of the total read length.

Fig. S2: Distribution of clusters of all EST-derived *Macrocystis pyrifera* ORFs with the brown algae split up by genus: *Laminaria*, *Fucus* (2 species), *Sargassum* and *Ectocarpus*. Annotations (when available) for clusters on the outside of the Venn are listed in Table S3.

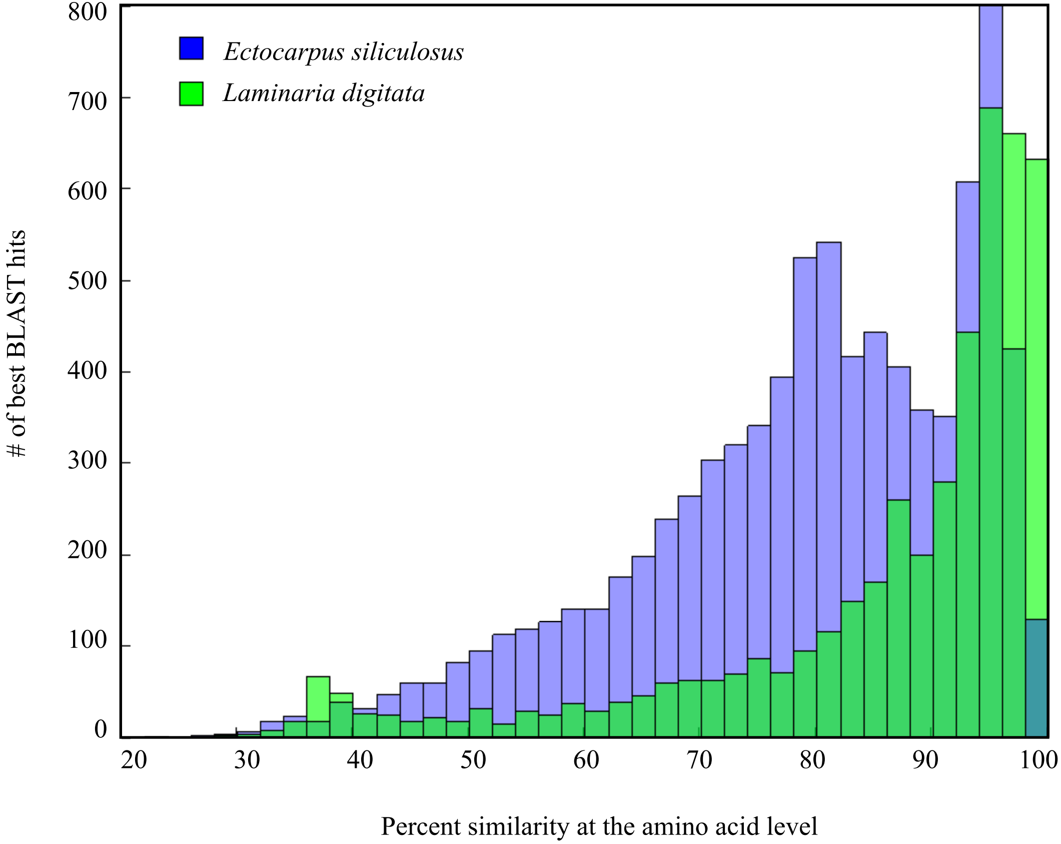

Ectocarpus siliculosus
Laminaria digitata
# of best BLAST hits
0
100
200
300
400
500
600
700
800
20
30
40
50
60
70
80
90
100
Percent similarity at the amino acid level

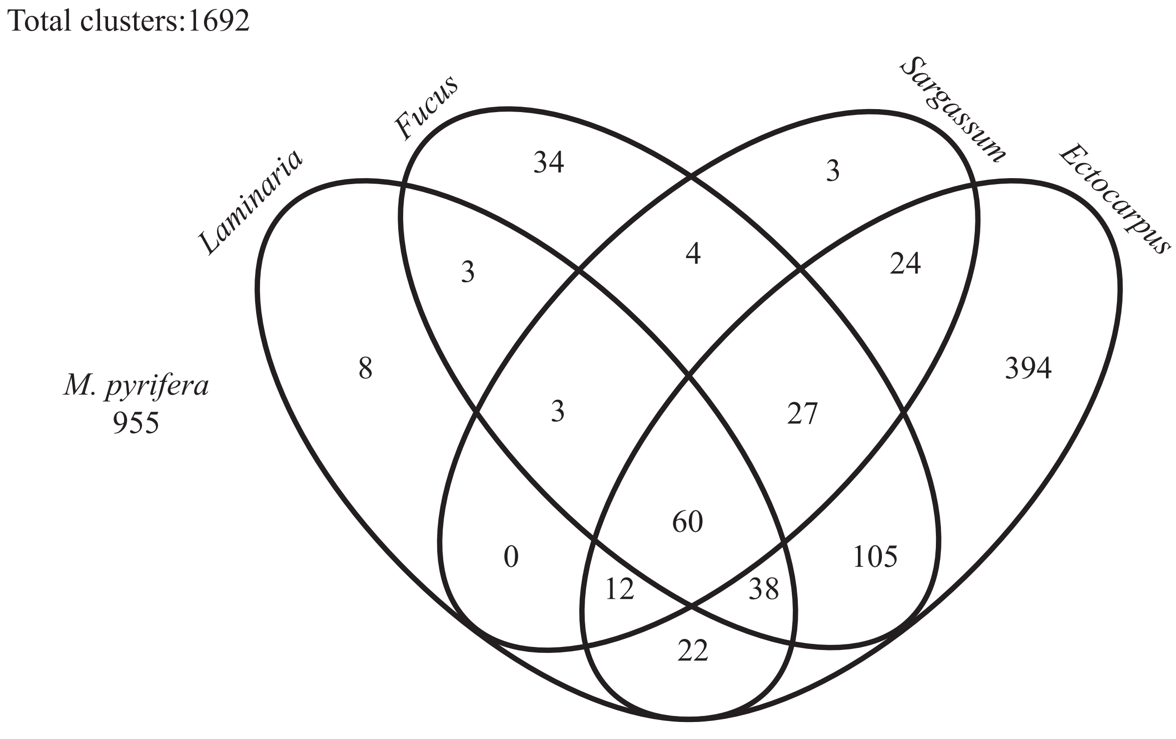

Total clusters:1692
Laminaria
Fucus
Sargassum
Ectocarpus
M. pyrifera
955
34
3
3
4
24
8
394
3
27
60
0
105
12
38
22

Table S1 *Macrocystis pyrifera* quantitative PCR targets, function, primer sets, and closest *Ectocarpus siliculosus* sequence match

Primer ID	Name	Function[1]	forward primer	reverse primer	Ectsi ortholog[2]	identities	%
18Smat	protein required for 18S rRNA maturation	housekeeper	gaacgtttaagcggttctcg	gtcacgggatttttcaccac	Esi0302_0026	123/128	97
ascperox	putative ascorbate peroxidase	stress response	gatgaggacgatctggagga	ccctgcagctaccctctgta	Esi0064_0006	88/103	86
aspg	Asparaginase	nitrogen metabolism	accggtgactgtaacgaagg	gtagacgccgcacctatctc	Esi0000_0456	45/57	79
ATPRXQ	putative peroxiredoxin	stress response	caggcatctgcaacttgtgt	tttcggtgaaaagcataccc	Esi0254_0022	47/52	91
bglu	betaglucosidase	carbohydrate metabolism	cttgtccagaacatcgctga	gatgcgcagacacgagaata	Esi0212_0019	64/91	71
CA	putative carbonic anhydrase	nitrogen metabolism	ccagcggtgtacatgagaga	gccttttccacgctgactac	Esi0174_0029	25/29	87
catperox	catalase/peroxidase	nitrogen metabolism	agctagaacaggtggcgaag	aaagagtctgcgccatgaac	Esi0612_0004	33/42	79
CPSIII	Carbamoyl-phosphate synthase	nitrogen metabolism	tatgacgacgtggtgcacat	acttgaccaggctggcatc	Esi0006_0183	32/50	64
f16ba	fructose-bisphosphate aldolase	carbohydrate metabolism	gctggtcgagctgagaattt	aacgacgagttcccgcttat	Esi0414_0009	71/82	87
fbp	Fructose-bisphosphatase	carbohydrate metabolism	acagccgtccgagattcat	ttcttgtcaccgagttgctg	Esi0512_0008	32/41	79
GCS	glycine decarboxylase	photosynthesis/carbon fixation	caacgagactttgacggaca	gtcgcagtgcttctcgtatg	Esi0046_0032	96/129	75
GH	Mannosyl-oligosaccharide glucosidase	carbohydrate metabolism	agcaatatgacgaccgaagc	gtcatcacaccagcacatcc	Esi0013_0087	52/59	89
GluSyn	Glutamate synthase	nitrogen metabolism	acgaccccaagggtaacttc	tagggaagagtccgtgtgct	Esi0103_0010	81/100	81
GluSynFe	Glutamate synthase (ferredoxin-dependent)	nitrogen metabolism	gatcgtgaaggggcttattg	ccagaatctcggcaggtact	Esi0000_0281	66/83	80
GPI	glucose-6-phosphate isomerase	carbohydrate metabolism	gaaccacgacgaactgatga	ctgtttcccgtgaacacctt	Esi0060_0128	109/137	80
GT	Glycosyl transferase	carbohydrate metabolism	tgctcattatatggcgcaag	acctcgtttgaaaaccgttg	Esi0201_0024	65/82	80
IF2A	translation initiation factor 2 alpha subunit	housekeeper	catagcctgcttcgtctcct	accaaggtctcttggcctct	Esi0216_0037	102/102	100
LHC1	Light harvesting complex protein	photosynthesis/carbon fixation	agggggatacagggtctcag	ccacgtgccagctaaagact	Esi0199_0055	29/31	94
LHC2	Light harvesting complex protein	photosynthesis/carbon fixation	aggaaattgacaacgcaacc	tgcctctgcttcacataacg	Esi0492_0007	95/125	76
LHC3	Light harvesting complex protein	photosynthesis/carbon fixation	gccgagttcttcgtgtcttc	tcctaacaggcggtggtaag	Esi0149_0020	54/65	84
LHC4	Light harvesting complex protein	photosynthesis/carbon fixation	cacgaggagctcaacaacaa	cacaaaagattcgacgagca	Esi0458_0016	138/140	99
LHC5	Light harvesting complex protein	photosynthesis/carbon fixation	cgtagcgtgaaaatgcagaa	accaactccctcttccacct	Esi0123_0002	68/71	96
LHC6	Light harvesting complex protein	photosynthesis/carbon fixation	agccggatgtagatggacag	gagggtacggagaaacacca	Esi0126_0039	90/115	79
LHC7	Light harvesting complex protein	photosynthesis/carbon fixation	agcttgtggatggcaagact	ataaaaacggtcagccaacg	Esi0009_0107	67/75	90
LHC8	Light harvesting complex protein	photosynthesis/carbon fixation	cgcctaccagacaagaaagc	ggtcaacatcctcggctaaa	Esi0085_0062	148/175	85
M1Pase	putative mannitol-1-phosphatase	carbohydrate metabolism	ggacaaaaccgcttctacga	agaccgagcggtccttaaat	Esi0080_0016	30/43	70
malDH	malate dehydrogenase	photosynthesis/carbon fixation	agtgcacgtttgttgactgc	agcttgctgaatccgtgaat	Esi0006_0195	79/98	81
NiR	nitrite reductase	nitrogen metabolism	aagcgctacggtcaacaact	accatttgagggaccaatca	Esi0249_0028	79/101	79
NR	Nitrate reductase	nitrogen metabolism	ccggagaaggtcgatgct	cttatccctggggtcgatct	Esi0006_0124	33/42	79
Ntrans	Nitrate high affinity transporter	nitrogen metabolism	cccctcatgaaggagatcaa	cgccgataaagaaacggata	Esi0278_0032	96/107	90
PFK	phosphofructose kinase	carbohydrate metabolism	gcaacagctttcgaccagat	caccgtcctgttattgacca	Esi0216_0030	112/124	91
PsbP	PsbP	photosynthesis/carbon fixation	cctggctgtgaacaagaaca	cgagtcgacgagagtcttga	Esi0254_0023	47/56	84
PyDH	pyruvate dehydrogenase (E1)	carbohydrate metabolism	tcgcactttgttggacgtag	ggatctgatacggcaaagga	Esi0122_0080	121/133	91
r5pi	ribose 5-phosphate isomerase	photosynthesis/carbon fixation	agggagaagatggtcgaggt	gacggtgttaagggtgtgct	Esi0126_0081	135/141	96
sperm	Spermine/spermidine synthase	stress response	aatgcctatggcttcacctg	ggtaggaaggcacggttgta	Esi0149_0059	119/143	84
UGP	putative UDP-glucose-pyrophosphorylase	carbohydrate metabolism	gtatccggcgtccactttta	atgttcggggaaaatgtcac	Esi0144_0004	92/99	93
updc	Uroporphyrinogen decarboxylase	photosynthesis/carbon fixation	cgggcatatccttaaccttg	gccccctccctatctaacac	Esi0207_0060	39/47	83
vperox	vanadium-dependent bromoperoxidase	stress response	gtacggcagggaagtagacg	tccatcctccagtgaacacc	Esi0009_0080	39/52	75

[1] Potential reference target primers tested (not selected for housekeeper normalization in this study): 18S ribosomal RNA gene, actin, alpha tubulin, beta tubulin, dynein heavy chain, eukaryotic elongation factor-1 B gamma, eukaryotic translation elongation factor 1 alpha, F-box zinc finger protein, glyceraldehyde 3-phosphate dehydrogenase, peptidyl-prolyl cis-trans isomerase, 40S ribosomal protein S26, ribosomal protein S7; and ubiquitin (x2).

[2] Closest tblastx *Ectocarpus siliculosus* sequence match, identities and percent

Table S2 Genomes and ESTs used in comparative cluster analysis and their sources

Species used in cluster analysis	sequence type	Total # ORFs	clustered ORFs	# clusters	Source	Project/Taxon ID
**Brown algae**						
Ectocarpus siliculosus	genome	16254	8615	5868	Univ. Ghent	EctoSi1009
Fucus serratus	EST	3648	2291	1165	NCBI	87148
Fucus vesiculosus	EST	1628	938	630	NCBI	49266
Sargassum binderi	EST	1116	692	519	NCBI	127580
Laminaria digitata	EST	1728	1026	521	NCBI	80365
Macrocystis pyrifera	EST	11844	3904	1692	this study	n/a
**Stramenopiles**						
Diatoms:						
Fragilariopsis cylindrus	genome	18077	10392	7233	JGI	Fracy1
Phaeodactylum tricornutum	genome	10402	7475	6232	JGI	Phatr2
Thalassiosira pseudonana	genome	11776	7369	5906	JGI	Thaps3
Pelagophytes:						
Aureococcus anophagefferens	genome	11501	6823	4621	JGI	Auran1
other Stramenopiles:						
Phytophthora infestans	genome	35028	24471	13219	NCBI	PRJNA49677
Phytophthora ramorum	genome	15605	13184	10161	NCBI	PRJNA12571
Phytophthora sojae	genome	18969	14718	10668	NCBI	PRJNA17989
**Green algae**						
Chlamydomonas reinhardtii	genome	17114	9489	7255	NCBI	PRJNA21061
Volvox carteri	genome	15544	9986	6874	JGI	Volca1
Micromonas sp. RCC299	genome	10056	7154	6633	JGI	MicpuN2
Micromonas pusilla CCMP1545	genome	10575	6794	6316	JGI	MicpuC2
Ostreococcus sp. RCC809	genome	7492	6824	6549	JGI	OstRCC809_2
Osterococcus tauri	genome	7725	6564	6233	JGI	Ostta4
Ostreococcus lucimarinus CCE9901	genome	7603	7057	6583	JGI	Ost9901_3
**Red algae**						
Porphyra haitanensis	EST	2364	897	750	NCBI	1262161
Porphyra yezoensis	EST	5344	1703	1343	NCBI	2788
Cyanidioschyzon merolae	genome	5014	2930	2615	Univ. Tokyo	merolae
Porphyridium purpureum	EST	13375	8938	4083	NCBI	35688

Table S3 Non-redundant annotations for novel clusters in Figures 2 and S2 with highlighted entries indicating annotations in common with those in Table S5 (the differentially expressed *M. pyrifera* ORFs).

**Phaeophyceae (not including *Macrocystis pyrifera* or *Ectocarpus*) clusters not seen in *Ectocarpus*, Stramenopiles, green algae or red algae (Fig. 2a)**<br>number of clusters = 543<br>number of clusters with annotation = 203
60s Acidic ribosomal protein
AAA domain (Cdc48 subfamily)
Actin
Acyl CoA binding protein
ADP-ribosylation factor family
AhpC/TSA family
Animal haem peroxidase
archaeal ribosomal protein S17P
ATP synthase
ATP synthase subunit C
C-terminal, D2-small domain, of ClpB protein
Caspase domain
Chlorophyll A-B binding protein
Cofilin/tropomyosin-type actin-binding protein
Coiled-coil domain containing protein (DUF2052)
Core histone H2A/H2B/H3/H4
Cyclophilin type peptidyl-prolyl cis-trans isomerase/CLD
Developmentally Regulated MAPK Interacting Protein
EF hand
EF-1 guanine nucleotide exchange domain
Elongation factor Tu C-terminal domain
Elongation factor Tu domain 2
Elongation factor Tu GTP binding domain
Eukaryotic-type carbonic anhydrase
FKBP-type peptidyl-prolyl cis-trans isomerase
FNIP Repeat
Fructose-1-6-bisphosphatase
Glyceraldehyde 3-phosphate dehydrogenase, C-terminal domain
Helicase conserved C-terminal domain
Hsp20/alpha crystallin family
Hsp70 protein
Hsp90 protein
Iron/manganese superoxide dismutases, C-terminal domain
Mago nashi protein
Mpv17 / PMP22 family
NAD binding domain of 6-phosphogluconate dehydrogenase
NADH:flavin oxidoreductase / NADH oxidase family
Nitrogenase component 1 type Oxidoreductase
Nucleoside diphosphate kinase
Opioid growth factor receptor (OGFr) conserved region
Outer membrane protein (OmpH-like)
Oxygen evolving enhancer protein 3 (PsbQ)
PAP2 superfamily
parallel beta-helix repeat
Peptidase family M41

Photosynthetic reaction centre protein
Photosystem I psaA/psaB protein
Proteasome subunit
Protein kinase domain
Protein of unknown function (DUF3308)
Pyridine nucleotide-disulphide oxidoreductase
Reverse transcriptase (RNA-dependent DNA polymerase)
Ribosomal family S4e
Ribosomal L27e protein family
Ribosomal L29 protein
Ribosomal L32p protein family
ribosomal L5P family C-terminus
ribosomal protein L10.e
Ribosomal protein L11, N-terminal domain
Ribosomal protein L11, RNA binding domain
Ribosomal protein L13
Ribosomal protein L13e
Ribosomal protein L14p/L23e
Ribosomal protein L16p/L10e
Ribosomal protein L18e/L15
Ribosomal protein L19e
Ribosomal protein L1p/L10e family
Ribosomal protein L23
ribosomal protein L24
ribosomal protein L29
Ribosomal protein L3
Ribosomal protein L31e
Ribosomal protein L32
Ribosomal protein L35
Ribosomal protein L35Ae
Ribosomal protein L36e
Ribosomal protein L6
Ribosomal protein L6e
Ribosomal protein L7Ae/L30e/S12e/Gadd45 family
Ribosomal protein S12
Ribosomal protein S17
Ribosomal protein S19e
Ribosomal protein S24e
Ribosomal protein S27a
Ribosomal protein S3, C-terminal domain
ribosomal protein S4
Ribosomal protein S4/S9 N-terminal domain
Ribosomal protein S6e
ribosomal protein S7
Ribosomal protein S7e
Ribosomal protein S7p/S5e
Ribosomal protein S8e
Ribosomal Proteins L2, C-terminal domain
Ribosomal Proteins L2, RNA binding domain
Ribosomal S13/S15 N-terminal domain
Ribosomal S17
Ribosomal S3Ae family
S1 RNA binding domain
S25 ribosomal protein

S4 domain
S4 domain:2RS4NT (NUC023) domain
Sec-independent protein translocase protein (TatC)
Serine incorporator (Serinc)
Synaptobrevin
Tetrahydrofolate dehydrogenase/cyclohydrolase, NAD(P)-binding domain
Tetratricopeptide repeat
Thiamine pyrophosphate enzyme, central domain
Translationally controlled tumour protein
Tubulin C-terminal domain
Ubiquitin family
WD domain, G-beta repeat

Macrocystis pyrifera clusters not seen in Phaeophyceae (including _Ectocarpus_), Stramenopiles, green algae or red algae (Fig. 2b)
number of clusters = 925
number clusters with annotation = 0

Macrocystis pyrifera clusters not seen in other members of the Phaeophyceae (_Laminaria, Fucus, Sargassum_ or _Ectocarpus;_ Fig. S2)
number of clusters = 955
number of clusters with annotation = 26

ABC transporter
ABC transporter transmembrane region
ABC-2 type transporter
Chaperonin 10 Kd subunit
Chlorophyll A-B binding protein
chromo' (CHRromatin Organisation MOdifier) domain
Cyclopropane-fatty-acyl-phospholipid synthase
Cytochrome b(C-terminal)/b6/petD
Cytochrome b(N-terminal)/b6/petB
Cytochrome c
Cytochrome C and Quinol oxidase polypeptide I
cytochrome c oxidase, subunit I
DEAD/DEAH box helicase
Glucose inhibited division protein A
Glutamine amidotransferase class-I
Glycoprotease family
Integrase core domain
Ketopantoate reductase PanE/ApbA C terminal
Met-10+ like-protein
metallohydrolase, glycoprotease/Kae1 family
Methyltransferase domain
Mitochondrial carrier protein
N-(5'phosphoribosyl)anthranilate (PRA) isomerase
Patched family
Phage integrase family
Proteasome non-ATPase 26S subunit
Protein of unknown function (DUF2488)

Protein tyrosine kinase
putative glycoprotease GCP
Reverse transcriptase (RNA-dependent DNA polymerase)
Ribonuclease HII
ribosomal protein L22
Ribosomal protein L22p/L17e
Ribulose-phosphate 3 epimerase family
RNA polymerase Rpb1, domain 3
RNA polymerase Rpb6
Tetratricopeptide repeat
Thiazole biosynthesis protein ThiG
transmembrane receptor (rhodopsin family)
tRNA uridine 5-carboxymethylaminomethyl modification enzyme GidA
YjeF homolog, C-terminus:4Carbohydrate kinase

Note: some clusters may have more than one annotation

Table S4 Continuation of Table 2: the top 31-100 ORFs with non-ribosomal pfam annotation ranked by total reads across the four libraries, the pfam description, the best species match, and significant differential expression patterns in January and July.

Sum	pfam description	taxon	Jan*	Jul*
78	BolA-like protein	*Ectocarpus siliculosus*		
75	Thioredoxin	*Ectocarpus siliculosus*		
71	Initiation factor 2 subunit family	*Ectocarpus siliculosus*		
70	HEAT repeat	*Ectocarpus siliculosus*		
67	FKBP-type peptidyl-prolyl cis-trans isomerase	*Ectocarpus siliculosus*		
66	Cyclophilin type peptidyl-prolyl cis-trans isomerase/CLD	*Ectocarpus siliculosus*		
66	GWT1	*Ectocarpus siliculosus*		
66	Protein of unknown function (DUF1336)	*Ectocarpus siliculosus*		+
65	Domain of unknown function (DUF377)	*Ectocarpus siliculosus*		+
64	Clathrin adaptor complex small chain	*Ectocarpus siliculosus*		
64	Mitochondrial glycoprotein	*Ectocarpus siliculosus*		
64	PCI domain	*Ectocarpus siliculosus*		
63	PAP_fibrillin	*Ectocarpus siliculosus*		
62	Endoplasmic reticulum vesicle transporter	*Ectocarpus siliculosus*		
61	ATPase family associated with various cellular activities (AAA)	*Ectocarpus siliculosus*		
61	Mitochondrial carrier protein	*Ectocarpus siliculosus*	+	
59	PsbP	*Ectocarpus siliculosus*		
57	Ubiquitin-conjugating enzyme	*Ectocarpus siliculosus*		
56	Glycine cleavage H-protein	*Ectocarpus siliculosus*		
56	Peptidase family M20/M25/M40‖Peptidase dimerisation domain	*Ectocarpus siliculosus*		
55	Reverse transcriptase (RNA-dependent DNA polymerase)	*Laminaria digitata*		
54	Acetyltransferase (GNAT) family	*Ectocarpus siliculosus*		+
54	Transcription factor S-II (TFIIS)	*Ectocarpus siliculosus*		
53	Quinolinate phosphoribosyl transferase, C-terminal domain	*Ectocarpus siliculosus*		
53	tRNA synthetases class I (W and Y)‖S4 domain	*Ectocarpus siliculosus*		
52	GDP dissociation inhibitor	*Ectocarpus siliculosus*		
52	Putative carnitine deficiency-associated protein	*Ectocarpus siliculosus*		
52	Ran-interacting Mog1 protein	*Ectocarpus siliculosus*		+
52	Thiazole biosynthesis protein ThiG‖Protein of unknown function (DUF2488)	*Crocosphaera watsonii* WH 8501		
51	Transaldolase	*Ectocarpus siliculosus*		
50	haloacid dehalogenase-like hydrolase	*Ectocarpus siliculosus*		
50	Iron-sulphur cluster biosynthesis	*Ectocarpus siliculosus*		
50	Peptide methionine sulfoxide reductase	*Ectocarpus siliculosus*		
49	3' exoribonuclease family, domain 13' exoribonuclease family, domain 2	*Ectocarpus siliculosus*		
49	Bucentaur or craniofacial development	*Ectocarpus siliculosus*		
49	CPSF A subunit region	*Ectocarpus siliculosus*		
49	Domain of unknown function (DUF298)	*Aureococcus anophagefferens*		
49	Eukaryotic translation initiation factor eIF2A	*Ectocarpus siliculosus*		
48	Isy1-like splicing family	*Ectocarpus siliculosus*		
48	Ketopantoate reductase PanE/ApbA C terminal	*Ectocarpus siliculosus*		
47	Tubulin C-terminal domain	*Ectocarpus siliculosus*		
46	Arginosuccinate synthase	*Laminaria digitata*		

46	GTPase of unknown function‖Elongation factor Tu GTP binding domain	*Ectocarpus siliculosus*		
46	NnrU protein	*Ectocarpus siliculosus*		
46	Shikimate / quinate 5-dehydrogenase‖Glutamyl-tRNAGlu reductase, dimerisation domain	*Ectocarpus siliculosus*		
46	tRNA synthetases class I (R)‖DALR anticodon binding domain	*Ectocarpus siliculosus*		
45	HMG (high mobility group) box	*Ectocarpus siliculosus*		
45	Peptidase M16 inactive domain	*Ectocarpus siliculosus*		
45	Rhomboid family	*Ectocarpus siliculosus*		
45	Uncharacterised P-loop hydrolase UPF0079	*Ectocarpus siliculosus*		
44	N2227-like protein	*Ectocarpus siliculosus*		
42	Enolase, C-terminal TIM barrel domain	*Ectocarpus siliculosus*		
42	Ergosterol biosynthesis ERG4/ERG24 family	*Ectocarpus siliculosus*		
41	Anticodon binding domain	*Fragilariopsis cylindrus*		
41	Complex I intermediate-associated protein 30 (CIA30)	*Ectocarpus siliculosus*	-	
41	Cwf15/Cwc15 cell cycle control protein	*Ectocarpus siliculosus*		
41	Dephospho-CoA kinase	*Ectocarpus siliculosus*		
41	Thioredoxin	*Ectocarpus siliculosus*		
41	Uncharacterized protein family UPF0027	*Ectocarpus siliculosus*		
40	TCP-1/cpn60 chaperonin family	*Ectocarpus siliculosus*		
40	Thiolase, C-terminal domain	*Ectocarpus siliculosus*		
38	SET domain	*Ectocarpus siliculosus*		
37	PQ loop repeat	*Ectocarpus siliculosus*		
35	Inositol monophosphatase family	*Ectocarpus siliculosus*		
35	Protein of unknown function	*Ectocarpus siliculosus*		
35	Pyridoxal-phosphate dependent enzyme	*Ectocarpus siliculosus*		
34	Protein of unknown function (DUF2470)	*Ectocarpus siliculosus*		+
34	Putative tRNA binding domain	*Ectocarpus siliculosus*		
33	Domain found in IF2B/IF5	*Ectocarpus siliculosus*		
33	RNA polymerase Rpb5, C-terminal domain	*Ectocarpus siliculosus*		

* Plus symbol(s) indicate higher expression in the surface; minus symbol(s) indicates higher expression at depth. The highest log-fold supported by a probability of 0.95 or greater is shown by the number of symbols; one symbol equals a fold change between 0.5 and 1.5, two symbols equals a fold change between 1.5 to 3.0, and three symbols equals a fold change > 3.0.

Table S5 Differentially expressed ORFs with pfam annotation and summed read counts across the four libraries, the best species match, and significant differential expression patterns in January and July.

Sum	pfam description	taxon	Jan*	Jul*
353	Photosynthetic reaction centre protein	*Odontella sinensis*	+++	+
210	Excisionase-like protein||Bacteriophage lambda integrase, N-terminal domain	*Phaeocystis globosa*	+++	
182	Chlorophyll A-B binding protein	*Fucus serratus*	+++	+
325	ATP synthase	*Ectocarpus siliculosus*	++	
137	GcpE protein	*Fucus vesiculosus*	+	
136	ATP synthase subunit D	*Ectocarpus siliculosus*	+	
134	RNA polymerase Rpb3/Rpb11 dimerisation domain	*Fucus serratus*	+	
116	Ribosomal protein L36||Ribosomal protein S13/S18	*Fucus serratus*	+	
82	Proteasome subunit	*Fucus vesiculosus*	+	+
61	Mitochondrial carrier protein	*Ectocarpus siliculosus*	+	
39	Ribosome recycling factor	*Ectocarpus siliculosus*	+	
131	Chaperonin 10 Kd subunit	*Nitzschia* I-146		+
97	Nucleoside diphosphate kinase	*Laminaria digitata*		+
83	Eukaryotic porin	*Ectocarpus siliculosus*		+
66	Protein of unknown function (DUF1336)	*Ectocarpus siliculosus*		+
65	Domain of unknown function (DUF377)	*Ectocarpus siliculosus*		+
54	Acetyltransferase (GNAT) family	*Ectocarpus siliculosus*		+
52	Ran-interacting Mog1 protein	*Ectocarpus siliculosus*		+
34	Protein of unknown function (DUF2470)	*Ectocarpus siliculosus*		+
30	ParA/MinD ATPase like||Protein of unknown function (DUF971)	*Ectocarpus siliculosus*		+
29	PAP_fibrillin	*Ectocarpus siliculosus*		+
18	Recombinase Flp protein	*Phaeocystis globosa*		+
132	Elongation factor Tu GTP binding domain	*Ectocarpus siliculosus*	-	++
713	Ribosomal protein S7e	*Sargassum binderi*	-	
324	Ribosomal protein L1p/L10e family	*Ectocarpus siliculosus*	-	
279	S25 ribosomal protein	*Laminaria digitata*	-	
240	Ribosomal protein L13e	*Ectocarpus siliculosus*	-	
232	Ribosomal protein S7p/S5e	*Ectocarpus siliculosus*	-	
212	Ribosomal protein L14p/L23e	*Laminaria digitata*	-	
191	Ribosomal protein S13/S18	*Ectocarpus siliculosus*	-	
180	Ribosomal protein S10p/S20e	*Ectocarpus siliculosus*	-	
109	DNA directed RNA polymerase, 7 kDa subunit	*Fucus vesiculosus*	-	
106	KH domain||Ribosomal protein S3, C-terminal domain	*Fucus serratus*	-	
99	Ribosomal protein L16p/L10e	*Ectocarpus siliculosus*	-	
97	Ribosomal protein L31e	*Ectocarpus siliculosus*	-	
86	Ribosomal protein L6	*Ectocarpus siliculosus*	-	
55	60s Acidic ribosomal protein	*Laminaria digitata*	-	
41	Complex I intermediate-associated protein 30 (CIA30)	*Ectocarpus siliculosus*	-	
243	Ribosomal protein S24e	*Laminaria digitata*		-
165	Ribosomal protein L34e	*Laminaria digitata*		-
136	Ribosomal L15	*Ectocarpus siliculosus*		-
65	Ubiquitin family||Ribosomal protein S27a	*Laminaria digitata*		-
129	Ribosomal S3Ae family	*Ectocarpus siliculosus*	-	-

* Plus symbol(s) indicate higher expression in the surface; minus symbol(s) indicates higher expression at depth. The highest log-fold supported by a probability of 0.95 or greater is shown by the number of symbols; one symbol equals a fold change between 0.5 and 1.5, two symbols equals a fold change between 1.5 to 3.0, and three symbols equals a fold change > 3.0.

Table S6 Light harvesting complexes identified from isotigs in *Macrocystis pyrifera*

Mp LHC ID	*Esi* ortholog	Dittami* ID	Family
LHC1	Esi_0199_0055	Esi_28	FCP
LHC2	Esi_0492_0007	Esi_47	FCP
LHC3	Esi_0149_0020	N/A	FCP
LHC4	Esi_0458_0016	Esi_42	FCP
LHC5	Esi_0123_0002	Esi_24	FCP
LHC6	Esi_0126_0039	Esi_25	Red
LHC7	Esi_0009_0107	Esi_03	LI818
LHC8	Esi_0085_0062	Esi_22	LI818

*Dittami et al 2010
